# Primary squamous cell carcinoma at ileostomy site. Two case reports and review of literature

**DOI:** 10.1002/ccr3.2931

**Published:** 2020-05-27

**Authors:** Murad Bani Hani, Bryan Butler, Daniel Leberer, Bashir Attuwaybi

**Affiliations:** ^1^ Colon and Rectal Surgery Sisters of Charity Hospital Buffalo NY USA; ^2^ State University of New York at Buffalo Buffalo NY USA

**Keywords:** complication, ileostomy, parastomal lesion, squamous cell carcinoma

## Abstract

SCC at ileostomy is rare, early diagnosis by sampling abnormal tissue around ileostomy is vital.

## Graphical abstract

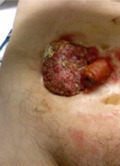

## 1 INTRODUCTION

A permanent end ileostomy is indicated in patients with diseases such as ulcerative colitis (UC), Crohn's disease or familial adenomatous polyposis (FAP) who require a total proctocolectomy and inability to restore intestinal continuity. Cancer at the ileostomy site is uncommon and pathology typically reveals adenocarcinomas, squamous cell carcinomas or lymphomas. Primary squamous cell carcinoma (SCC) at the ileostomy site is extremely rare.

## 2 CASE REPORTS

**FIGURE 1:**
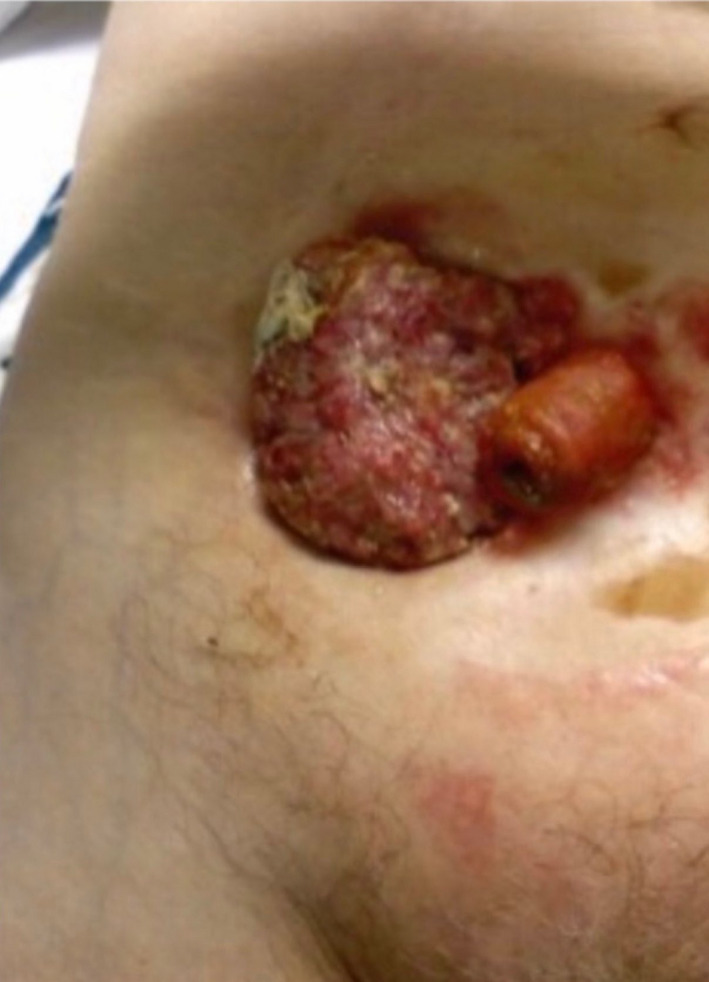
Parastomal adenocarcinoma, gross view (First patient)

**FIGURE 2:**
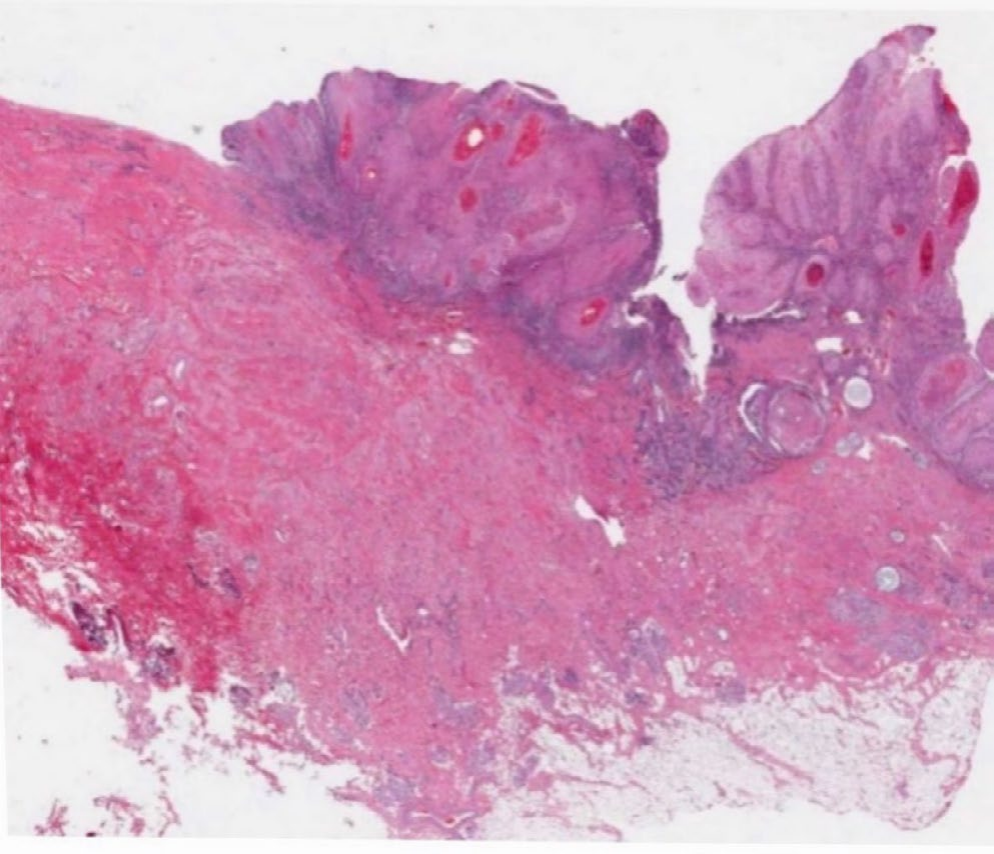
Parastomal adenocarcinoma, microscopic view (First patient)

The first patient was a 78‐year‐old female with history of UC who underwent a total proctocolectomy with end ileostomy 62 years prior to evaluation. She presented with an exophytic friable mass lateral to the ileostomy (Figure 1). A biopsy performed in the office showed a well‐differentiated squamous cell carcinoma (Figure 2). CT of chest, abdomen, and pelvis had no evidenced of distant metastasis.

She was treated with wide local excision of the tumor with en bloc resection of the ileostomy and creation of a new ileostomy in a different location in the left lower quadrant.

Final pathology revealed an invasive, moderately differentiated squamous cell carcinoma measuring 6.8 × 5.5 × 3.5 cm. There was squamous cell carcinoma in situ in the small bowel mucosa of the ileostomy. All surgical margins were negative for malignancy.

**FIGURE 3:**
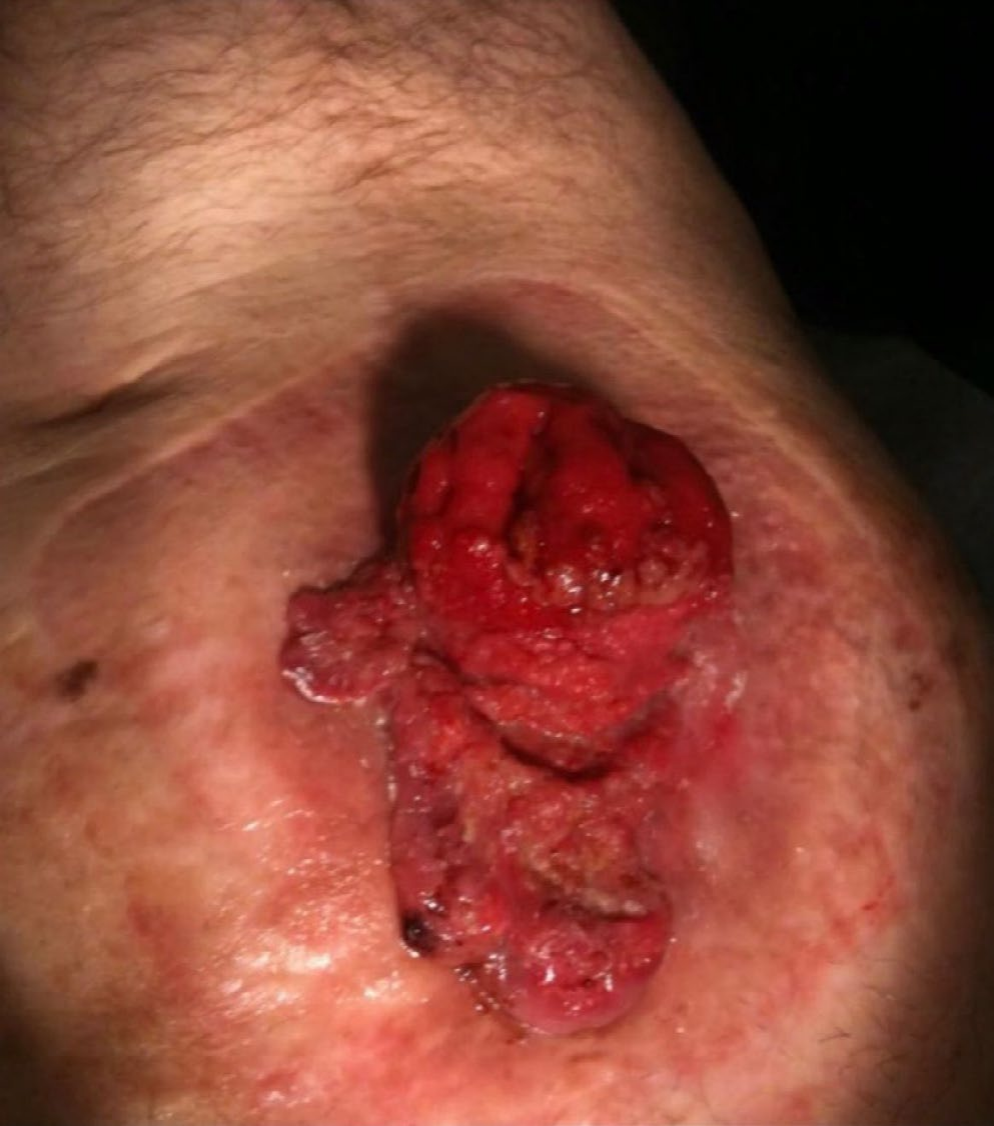
Parastomal adenocarcinoma, gross view (Second patient)

The second patient was a 76‐year‐old male with a history of UC who underwent a total proctocolectomy with end ileostomy 30 years prior to evaluation. He presented with a bleeding flat ulcerated mass at the inferolateral aspect of the ileostomy with irritation of the surrounding skin (Figure 3). A biopsy confirmed a squamous cell carcinoma. He was treated with a wide local excision of the tumor with en bloc resection and relocation of the ileostomy.

## 3 DISCUSSION

Primary squamous cell carcinoma at the ileostomy site is an extremely rare tumor. Literature search including PubMed, Google, and Medline, yielded only 6 reported cases prior to our report.^1^

The first case reported in 1966 was in a 76‐year‐old woman, 53 years after a total proctocolectomy for UC.^2^ In 2002, Ramanujam et al^3^ reported another case with literature review from 1966 through 2005. They found only three cases reported, including the one they presented. Since then, Farshid and colleagues published a case report and another literature review in which they reported a total of 6 cases of SCC at the ileostomy site.^1^

The available literature suggests that SCC is a late complication that is usually seen many years after creation of a permanent ileostomy. The earliest presentation was 26 years after ileostomy creation.^1^, ^2^

Table 1 below summarizes the patients' demographics, initial diagnoses, timing of presentation after ileostomy creation and the treatment of squamous cell carcinoma at the ileostomy site.

**TABLE 1:** Squamous cell carcinoma at ileostomy cases

Demographic	Primary diagnosis	Initial surgery	Timing after ileostomy (y)	SCC treatment
78 y F	Ulcerative colitis	Proctocolectomy, end ileostomy	62	Radical excision and re‐siting of stoma
76 y M	Ulcerative colitis	Proctocolectomy, end ileostomy	30	Radical excision and re‐siting of stoma
76 y M (1)	Ulcerative colitis	Total colectomy, end ileostomy	54	Palliative radiotherapy
70 y F (1,2)	Crohn's disease	Proctocolectomy, end ileostomy	26	Radical excision and re‐siting of stoma
67 y M (1,3)	Ulcerative colitis	Proctocolectomy, end ileostomy	44	Radical excision and re‐siting of stoma
66 y M (1,4)	Ulcerative colitis	Proctocolectomy, end ileostomy	41	Radical excision and re‐siting of stoma
76 y F (1,5)	Ulcerative colitis	Proctocolectomy, end ileostomy	51	Radical excision and re‐siting of stoma
80 y M (1,9)	Transitional CC	Radical cystoprostatectomy ileal conduit	27	Soft tissue excision. abdominal wall reconstruction

The main etiology is believed to be chronic irritation^1^, ^4^, ^5^, ^6^, ^7^ secondary to toxins in the stool and/or urine, or recurrent skin infections over the years. Both situations could cause a localized chronic response leading to metaplasia that could subsequently progress to carcinoma.^1^, ^8^

Symptoms are usually skin irritation, bleeding, abnormal growth around ileostomy site, and difficulty with placement of the ileostomy appliance.

Workup should include a biopsy to confirm the diagnosis and a CT chest/abdomen/pelvis to rule out distant metastasis. Treatment is surgical and includes radical en bloc excision of the tumor and ileostomy. Creation of a new ileostomy at a different location is also advisable.

## 4 CONCLUSION

Squamous cell carcinoma at the ileostomy site is an extremely rare tumor. The wound care/ostomy nurse and the surgeon should be aware of this late complication and annual exam of the ileostomy is advised. If a patient presents with symptoms of skin irritation, bleeding at or around the ileostomy, a non‐healing ulcer, or any abnormal growth, a thorough examination should be performed which mandates removal of the stoma appliance. Any suspicious lesions should be considered for biopsy.^1^, ^9^ It is vital to stress the importance of appropriate ostomy care to avoid chronic irritation/inflammation.

Treatment of squamous cell carcinoma at the ileostomy is a wide excision with en bloc resection of the ileostomy and creation of a new ileostomy placed in a different location.^9^ Prognosis is good if negative surgical margins are achieved.

## CONFLICT OF INTEREST

None declared.

## AUTHOR CONTRIBUTION

Dr Bani Hani and Dr Attwaybi: conceived of the presented idea. All authors discussed the content of the manuscript and contributed to the final manuscript. Dr Bani Hani: wrote the manuscript with support from Dr Attuwaybi, Dr Butler, and Dr Leberer. Dr Bani Hani and Dr Attuwaybi: completed the literature review. Dr Leberer and Dr Butler: interviewed the patients, collected their data, and asked permission to share their cases clinical photos. All authors helped reviewing the final version of the manuscript and edit it as needed.
